# Dynamic allocation and transfer of non-structural carbohydrates, a possible mechanism for the explosive growth of Moso bamboo (*Phyllostachys heterocycla*)

**DOI:** 10.1038/srep25908

**Published:** 2016-05-16

**Authors:** Xinzhang Song, Changhui Peng, Guomo Zhou, Honghao Gu, Quan Li, Chao Zhang

**Affiliations:** 1 The Nurturing Station for the State Key Laboratory of Subtropical Silviculture, Zhejiang A&F University, Lin’an, 311300, China; 2 Department of Biology Sciences, Institute of Environmental Sciences, University of Quebec at Montreal (UQAM), Case postale 8888, Succursale Centre-Ville, Montréal, QC, H3C 3P8, Canada; 3 Laboratory for Ecological Forecasting and Global Change, College of Forestry, Northwest Agriculture and Forest University, Yangling 712100, China

## Abstract

Moso bamboo can rapidly complete its growth in both height and diameter within only 35–40 days after shoot emergence. However, the underlying mechanism for this “explosive growth” remains poorly understood. We investigated the dynamics of non-structural carbohydrates (NSCs) in shoots and attached mature bamboos over a 20-month period. The results showed that Moso bamboos rapidly completed their height and diameter growth within 38 days. At the same time, attached mature bamboos transferred almost all the NSCs of their leaves, branches, and especially trunks and rhizomes to the “explosively growing” shoots via underground rhizomes for the structural growth and metabolism of shoots. Approximately 4 months after shoot emergence, this transfer stopped when the leaves of the young bamboos could independently provide enough photoassimilates to meet the carbon demands of the young bamboos. During this period, the NSC content of the leaves, branches, trunks and rhizomes of mature bamboos declined by 1.5, 23, 28 and 5 fold, respectively. The trunk contributed the most NSCs to the shoots. Our findings provide new insight and a possible rational mechanism explaining the “explosive growth” of Moso bamboo and shed new light on understanding the role of NSCs in the rapid growth of Moso bamboo.

As the main product of plant photosynthesis, carbohydrates can largely be partitioned into structural carbohydrates (SCs) and non-structural carbohydrates (NSCs) according to their roles^1^,^2^,^3^. SCs, including lignin, cellulose, hemicelluloses and pectin, are mainly used for the structural growth of plants^4^. The NSC pool is the sum of soluble sugars and starch^5^,^6^,^7^. As major components of the carbon reserves, NSCs can be remobilized for use and play a fundamental role in plant germination, growth, reproduction, defense and survivorship under stress^1^,^2^,^3^,^8^. NSCs can provide a temporary source of carbon when current photosynthesis cannot meet the immediate carbon demands of the plant. Once mobilized, NSCs can be used to support metabolism, structural growth, defense, and reproduction^9^,^10^,^11^.

Previous investigations have usually focused on the responses of NSCs to drought stress^12^,^13^,^14^, treeline^5^,^15^, and flushing or bud break^16^,^17^,^18^. Recent studies have suggested that a large fraction of a tree’s annual Carbon budget is allocated to the NSC pool^19^,^20^. The NSC pool is depleted when demand exceeds supply, such as when metabolism and growth requirements are high or when the production of photoassimilates is limited, and is refilled when the supply exceeds demand, such as when metabolism and growth requirements are low^1^,^7^,^21^,^22^. NSCs are the most important Carbon reserves in the tissues of deciduous and evergreen tree species and serve as carbon sources to satisfy the carbon demand during the flushing of new leaves and shoots in spring^16^,^17^,^18^,^23^. The NSC concentration has been observed to decrease sharply in response to the increased carbon demands during flushing or bud break^16^,^17^,^18^. The lack of photosynthetically active tissues at bud break results in broad-leaved trees that are dependent on last season’s carbon reserves for flushing^24^. The high carbon demand during bud break may exceed the carbon supplied by current photosynthesis in evergreen species^25^ and also depend partly on the NSC pool. Fast-growing plants have high carbon demands during periods of fast growth. However, the NSC dynamics during such fast growth periods have received little attention.

Moso bamboo (*Phyllostachys pubescens* Mazel ex H. de Lehaie) forests are currently the most important source of non-wood forest products in China, covering an area of 3.87 million ha, representing 70% of the country’s bamboo forest area and 80% of the global distribution of *P. pubescens*^26^,^27^. As a woody rhizomatous plant, Moso bamboo is a monopodial giant bamboo with a mean height of 10–20 m and diameter at breast height (DBH) of 8–16 cm, and it is well-known for its fast growth rate^28^,^29^. In contrast to the arborous species, Moso bamboos can complete their growth in height and diameter within 35–40 days after shoots emerge from the soil in the spring. Afterwards, the height, diameter and volume remain unchanged because of the scarce secondary cambium, and the bamboo begins to slowly accumulate dry matter^29^,^30^. Our previous study observed the mean height of each shoot of young bamboo to increase 28 times (from 0.46 to 12.83 m), whereas the mean carbon storage amount increased 45 times (from 0.04 to 1.82 kg) in 28 days (from April 13 to May 10)^29^. Ueda^31^ also reported that Moso bamboo shoots can even elongate by up to 1 m per day during the period of fastest growth. The rapid growth of Moso bamboo is called “explosive growth” by local people. However, the underlying mechanism of this “explosive growth” remains unclear and mysterious.

The recruitment of new bamboo is carbohydrate-dependent^28^. However, during the “explosive growth period (EGP),” there is no photosynthesis because the new bamboos begin to expand their branches and leaves only after completing their growth in height^28^,^29^. Therefore, it was speculated that the carbohydrates and nutrients needed for the construction of new bamboo are provided by other attached mature bamboos via underground rhizomes^29^,^32^ (Fig. 1). However, this hypothesis lacked support from direct evidence and needed to be tested by field observations. To our knowledge, no previous studies have been conducted to measure the NSC dynamics of Moso bamboo and its potential relationship with seasonal growth dynamics.

Here, we present a series of observations and experiments that examine the NSC changes in Moso bamboo shoots and attached mature bamboos during fast growth of bamboo shoots to test the hypothesis that the carbohydrates (in the form of NSCs) needed for the construction and metabolism of bamboo shoots during EGP are provided by attached mature bamboos via underground rhizomes.

## Results

### Dynamics of height and biomass of shoots - young bamboos

**Figure 2:**
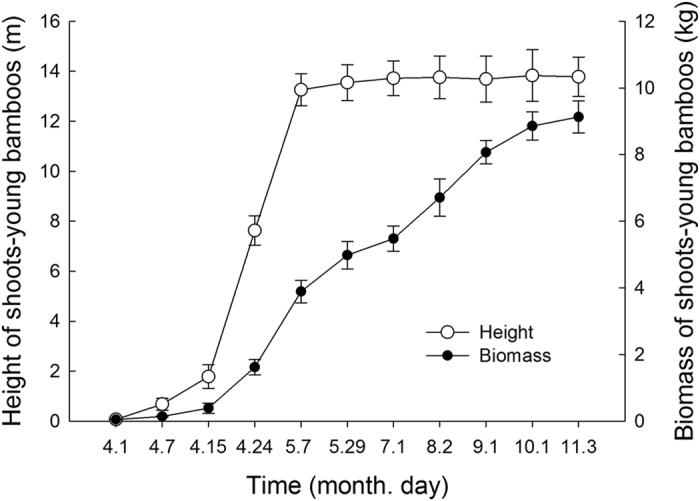
Height growth and biomass accumulation of Moso bamboo shoots: young bamboos after shoot emergence. The bar denote the standard error (n = 3). The same notation is used below.

In approximately the first 15 days after Moso bamboo shoots emerge, the height growth and biomass accumulation of the shoots were relatively slow (Fig. 2). Approximately 15 days after emergence, however, the shoot height showed a sharp increase from 1.78 m on April 15 to 13.26 m on May 7, combined with a rapid biomass accumulation from 0.39 kg to 3.89 kg. After that point, the height growth tended to stop, but the rapid accumulation of biomass continued until October 1 and then tended to slow.

### NSCs dynamics of shoots - young bamboos

**Figure 3:**
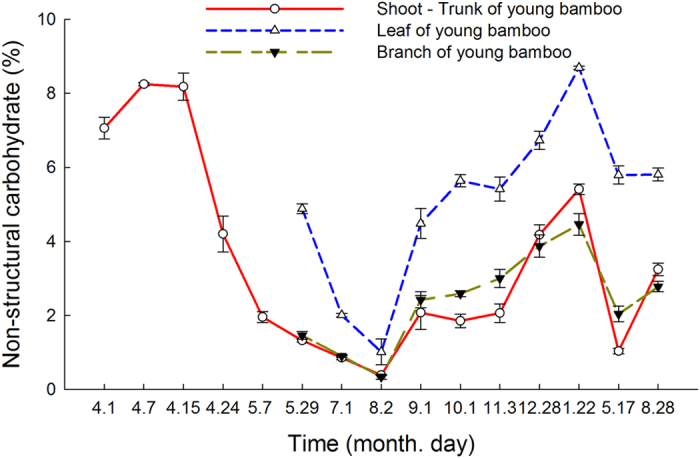
Dynamics of the non-structural carbohydrate (NSC) content of Moso bamboo shoots: young bamboos after shoot emergence.

In approximately the first 7 days after Moso bamboo shoots emerged, the NSC content of the bamboo shoots showed an increase to 8.25% (Fig. 3). However, approximately 15 days after emergence, the NSC content of the shoots sharply declined. After 22 days (May 7), this sharp decline tended to slow. The NSC content reached a minimum on August 2 (approximately 4 months after shoot emergence) and then began to increase again until the following January. Then, the NSC content declined again and reached a minimum in May, followed by rising again. On May 29 of 2014, new leaves and branches had grown out from the Moso bamboo shoots, indicating that the bamboo shoots had become young bamboos. The NSC content of the leaves and branches showed the same changing trend as the trunks of the young bamboos, but the NSC content of the leaves was always significantly higher than that in the branches and trunks at every sampling (*P* < 0.05).

### NSC dynamics of mature Moso bamboos

**Figure 4:**
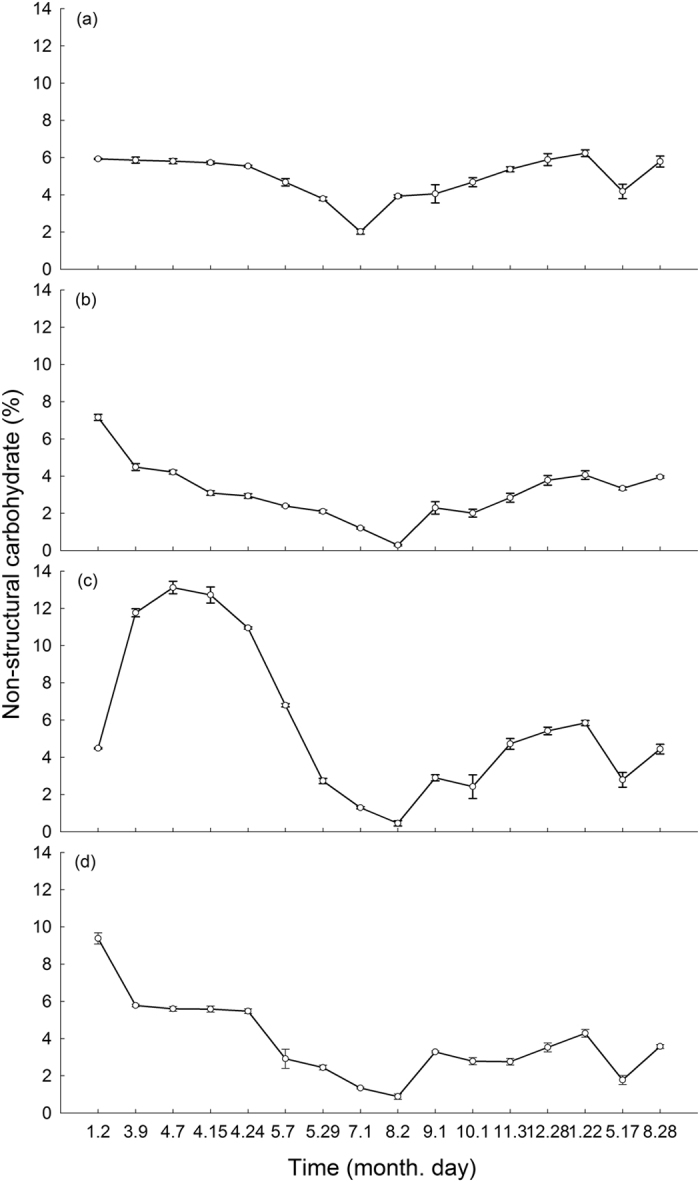
Dynamics of the non-structural carbohydrate (NSC) content of each part of attached mature Moso bamboos over a 20-month period (from January 2014 to August 2015). (**a**) leaf; (**b**) branch; (**c**) trunk; (**d**) rhizome. Different lowercase letters indicate significant differences (*P* < 0.05) in the NSC content among samplings.

The changes in the NSC content of the leaves, branches, trunks and rhizomes of mature bamboos attached to each sampled shoot are shown in Fig. 4. The NSC content of the leaves began a rapid and significant decline after April 24 and reached its lowest levels on July 1, having been reduced by 1.5 fold, and then gradually rose and approached the levels prior to shoot emergence until the following January. The NSC content then began to decline again and reached a minimum in May, followed by rising again (Fig. 4a). The NSC content of the branches declined continuously from January 2 to August 2 with a large change from 7.16% to 0.29%, a reduction of 23 fold, and then gradually rose and declined repeatedly, following the same changing pattern as the leaves (Fig. 4b). The NSC content of the trunks showed a rapid increase before April 7, from 4.48% to 13.12%, and then declined until August 2 with a minimum of 0.45%, a reduction of 28 fold (Fig. 4c). In particular, the NSC content declined sharply by 4 fold from 12.72% to 2.73% in the 44 days from April 15 to May 29. After August 2, the NSC content of the trunks also began to gradually increase and then decline repeatedly, following the same changing pattern as the leaves and branches. After a temporary decline, the NSC content of the rhizomes linking the shoots and mature bamboos showed a stable period from March 9 to April 24 (Fig. 4d). Then, the NSC content also underwent a fast decline until August 2 with a minimum of 0.88%, a reduction of 5 fold. After August, similar to the trunks and branches, the NSC content of the rhizomes also exhibited the same fluctuating pattern.

## Discussion

Moso bamboo shoots rapidly completed their height growth within 38 days after emerging and reached an average height of 13.26 m, which was consistent with the previous studies^29^,^31^. The biomass also accumulated rapidly during the EGP (Fig. 2). There were no new leaves growing from the shoots, and therefore photoassimilates could not be produced during this period. Thus, the composition of the shoot biomass could come from only attached matured bamboos through underground rhizomes^29^,^32^. Our observations showed that the NSC content of the leaves, branches, and especially the trunks and rhizomes of attached mature bamboos declined sharply during this period (Fig. 4). These missing NSCs were thought to be re-allocated and transferred to the emerging shoots through the underground rhizomes, then transformed and utilized in the structural growth (i.e., height growth) and metabolism of the shoot. It has been observed that the coarse fiber concentration of the shoots increases during rapid growth^33^. In our study, the trunks accounted for a higher proportion (79.69%) of aboveground biomass than the leaves (7.61%) and branches (12.70%) (Table 1), which indicated that the trunks contributed the most NSCs to the shoots. The sharp decline in the NSC content of the shoots from the high levels at emergence also supported this inference (Fig. 3). Ding *et al*.^34^ observed that the sap flow rate of trunks of mature Moso bamboos showed a pronounced increase from the middle of April to the end of May. This change in the rate of sap flow, the transport carrier of NSCs, also supported the hypothesis.

After height growth was completed (approximately May 7), new leaves and branches began to grow out from the young bamboo, and this period continued until the middle-to-end of July^35^,^36^. The structural growth and metabolism of the leaves and branches, as well as the strengthening of the young bamboos, also consume large amounts of NSCs^37^. These growth processes led to a continuous decline in NSCs in both young bamboos and attached mature bamboos after May 7, at which time the height growth was completed (Figs 3 and 4). Moreover, the NSCs were first distributed to leaves to maximize the photosynthetic capacity to independently supply carbohydrates for the young bamboo itself as early as possible. Therefore, the NSC content of the leaves was always higher than that in the branches and trunks of the young bamboos (Fig. 3). It was observed that the NSC content of Scots pine (*Pinus sylvestris* L.) decreased during bud break^16^,^17^. Landhäusser and Lieffers^18^ also observed the sharp reduction of NSCs in the branches and stems of *Populus tremuloides* clones during leaf flushing. It was also reported that the bud break of broad-leaved trees depended on last season’s carbon reserves^24^. Schädel *et al*.^25^ demonstrated that the high carbon demand during bud break in evergreen species also partly depended on the NSC pool.

After August 2, the NSC pool in the leaves, branches and trunks of young bamboos gradually recovered (Fig. 3). It has been reported that after leaves sprouted from the young bamboos, the content of chlorophyll a and b in the leaves increased quickly until October, indicating a rapid enhancement of the photosynthetic capacity of the leaves of the young bamboos^38^,^39^. After August, photoassimilation production gradually exceeded the demands of the young bamboos for carbohydrates, and thus the NSC content started to rise, which may reasonably explain the NSC recovery in the young bamboos. Then, the attached mature bamboo no longer needed to transfer NSCs to the young bamboo. At that time, in summer, the highest daily photosynthetic production of mature bamboo during the year also occurred^40^. Therefore, the NSC content of each part of the mature bamboo also gradually increased from then on (Fig. 4). In fact, the NSC content of the leaves of mature bamboos began to rise after July 1, indicating that the recharging of the NSC pools in the leaves, the most important photosynthetic organ, had begun earlier.

The leaves of young bamboo all fall in the following spring, and then new leaves with a lifespan of two years grow^32^. Therefore, the sampled leaves of both young and mature bamboo on May 17, 2015 were senescent leaves, which may explain the sharp decline in the NSC content of leaves in May 2015. This defoliating period in spring also resulted in the decline of the NSC content in branches and trunks. Then, as the new leaves grew, the NSC content began to increase again.

## Conclusion

Within 38 days after emerging, Moso bamboo shoots completed their “explosive growth” and reached an average height of 13.26 m and biomass of 3.89 kg. During this period, almost all the NSC content of the leaves, branches, and especially trunks and rhizomes of attached mature bamboos was re-allocated and transferred to the “explosively growing” shoots via underground rhizomes for both the structural growth and the metabolism of the shoots. The transfer process of NSCs from attached mature bamboos to shoots (young bamboos) stopped when the leaves of the young bamboos could provide enough carbohydrates (photoassimilates) to meet the immediate carbon demands of the young bamboos. Afterward, the depleted NSC pool of the attached mature bamboos gradually recovered.

## Materials and Methods

### Study site

The study site was located in Qingshan Town, Lin’an city (30°14′N, 119°42′E), Zhejiang Province, China. This area has a monsoonal subtropical climate with four distinct seasons. The mean annual precipitation is 1,420 mm, and the mean annual temperature is 15.6 °C, with maximum and minimum temperatures of 41.7 °C and −13.3 °C, respectively. The area receives an average of approximately 1,847 hours of sunshine per year and an average of 230 frost-free days per year.

The Moso bamboo plantations were originally established in the late 1970 s from native evergreen broadleaf forest in sites of similar topography (southwest slope of approximately 6 degrees) and soil type. The soils are classified as Ferrisols derived from granite^41^. Management practices, including annual fertilization, plowing, and weeding with herbicide, have been conducted since 2001. In September of each year, fertilizer compounds (N:P_2_O_5_:K_2_O: 15:6:20, 450 kg ha^−1^) are distributed and followed by a deep plowing to 0.3 m. Twelve understory species, dominated by *Viola prionantha*, achieve a mean height of 0.1 m, a forest floor coverage of 7%, and a total herbal biomass of 16.3 kg ha^−1^.

### Experimental design and sampling

The growth of the shoots of Moso bamboo is strongly seasonal. Shoots are usually initiated from solitary lateral buds on below-ground rhizomes every summer (June–August), then begin to emerge aboveground at the end of the following March and attain their full size approximately two months after emergence. By the middle-to-end of May, the shoots reach the top of the canopy and develop to their full height and DBH, becoming young bamboos^28^,^29^. Moreover, Moso bamboo plantations are characterized by alternating high- and low-recruitment years in the shoots due to long-term management practices^29^,^35^,^42^. In the present Moso bamboo stands, the recruitment of shoots is high in every even-numbered year (2012, 2014, and so on) and low in every odd-number year (2011, 2013, and so on). To observe the changes in the NSCs of bamboo shoots and attached mature bamboos during explosive growth, we performed a 20-month field investigation experiment beginning in a high-growth year, from January 2014 to August 2015.

In November 2013, three plots of 30 × 30 m were established. The initial stand and soil characteristics are summarized in Table 2. Twenty two-year-old bamboos near the mean DBH were selected and marked in each plot. Before the shoots emerged on April 1, two samplings were performed on January 2 and March 9, respectively. One marked bamboo in each plot was cut during every sampling, the fresh weight of the sample was weighed and subsamples of leaves, branches, trunks and attached rhizomes were collected in insulated cases at 4 °C for transfer to the laboratory as soon as possible. After shoot emergence on April 1, all the shoots emerging on April 1 and 2 were marked for the next sampling. Samples of the marked shoots and attached marked bamboos were collected on April 7, April 15, April 24, May 7, May 29, July 1, August 2, September 1, October 1, November 3, December 28, January 22, May 17, and August 28. During every sampling, a marked shoot with normal growth was first selected in each plot, and then a markedly attached bamboo was also selected along the underground rhizome, which was mainly distributed in the top 30 cm of the soil. One selected shoot, attached rhizome and two-year-old bamboo were cut during every sampling in each plot. The height of the shoot was recorded, and the fresh weights of the shoot, rhizome and bamboo samples were measured. The subsamples of shoots, attached rhizomes, leaves, branches and trunks of the attached bamboo were also weighed and collected in insulated cases at 4 °C for transfer to the laboratory as soon as possible. Subsamples were heated at 105 °C for 30 minutes, then oven-dried at 65 °C to a constant weight to analyze the water content and NSC content of the fresh samples. After November 3, only 200 g for each sample of leaves, branches and trunks was collected from one marked young and mature bamboo pair by tree trimmer, without cutting. Samples were heated at 105 °C for 30 minutes, then oven-dried at 65 °C to a constant weight. It is important to note that after completing their height growth at the middle-to-end of May, the bamboo shoots become young bamboos and begin to grow branches and leaves. Thus, during the last several samplings after May 7, the leaves and branches of young bamboos were also sampled.

The oven-dried samples were ground with a grinder (DFT-50 A, Wenling LINDA Machinery Co. Ltd., China). The NSC concentration was determined using an improved colorimetric method^43^,^44^.

### Data and statistical analysis

One-way analysis of variance (ANOVA) and least significant difference (LSD) tests were used to determine the statistical significance of the differences in the NSC content of leaves, branches, trunks and rhizomes among sampling events. The data were checked and satisfied the assumptions of homogeneity of variance. These analyses were conducted using SPSS (Statistical Package for the Social Sciences) 16.0 for Windows software (SPSS Inc., Chicago, Illinois).

## Additional Information

**How to cite this article**: Song, X. *et al*. Dynamic allocation and transfer of non-structural carbohydrates, a possible mechanism for the explosive growth of Moso bamboo (*Phyllostachys heterocycla*). *Sci. Rep*. **6**, 25908; doi: 10.1038/srep25908 (2016).

## Figures and Tables

**Figure 1:**
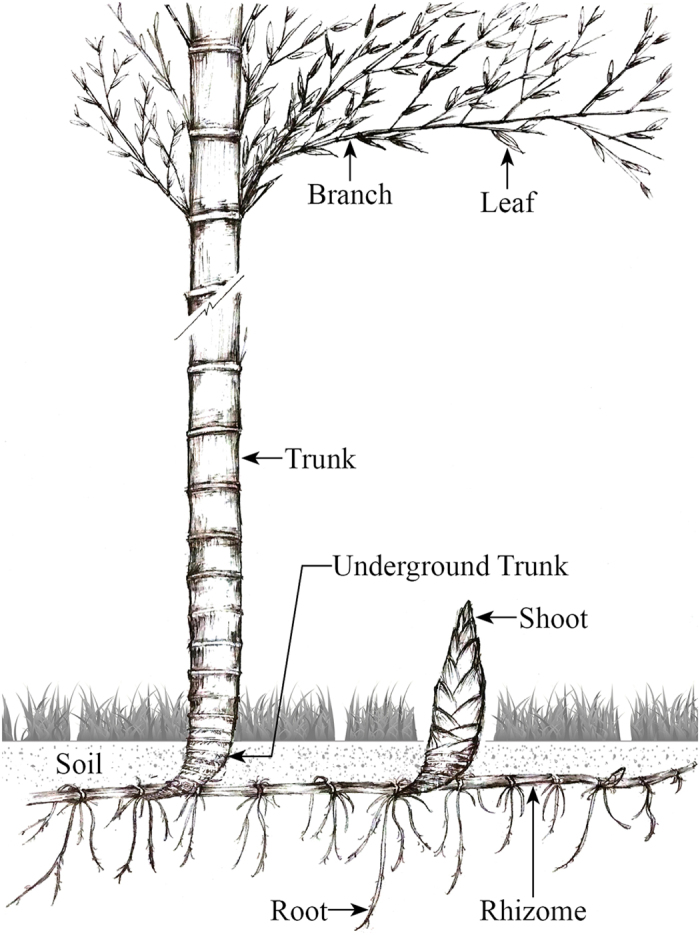
Sketch of relationships among shoot, mature Moso bamboo and rhizome. The map was designed and created by Xinzhang Song and Chao Zhang using Adobe Photoshop 7.0 (Adobe Systems Software Ireland Ltd).

**Table 1:** Aboveground biomass allocation and proportion of mature bamboos (mean ± SD, *n* = 36).

Organ	Biomass (kg)	Percentage (%)
Leaf	0.94 ± 0.52	7.61
Branch	1.57 ± 0.68	12.70
Trunk	9.85 ± 2.17	79.69

**Table 2:** Initial stand and soil characteristics of the study sites in the Moso bamboo forest (mean ± SD, *n* = 3).

Stand density (trees ha^−1^)	DBH (cm)	SBD (g cm^−3^)	SOC (mg g^−1^)	TN (mg g^−1^)	AN (mg g^−1^)	TP (mg g^−1^)	AP (mg g^−1^)	Soil pH
3428 ± 283	10.42 ± 0.15	1.05 ± 0.08	22.87 ± 0.3	1.24 ± 0.04	0.11 ± 0.004	0.45 ± 0.02	0.003 ± 0.000	4.51 ± 0.04

DBH: diameter at breast height; SBD: soil bulk density; SOC: soil organic C; TN: soil total N; AN: available nitrogen; TP: soil total P; AP: available phosphorus.
